# A CRISPR/Cas9 and Cre/Lox system-based express vaccine development strategy against re-emerging Pseudorabies virus

**DOI:** 10.1038/srep19176

**Published:** 2016-01-18

**Authors:** Xun Liang, Leqiang Sun, Teng Yu, Yongfei Pan, Dongdong Wang, Xueying Hu, Zhenfang Fu, Qigai He, Gang Cao

**Affiliations:** 1 State Key Laboratory of Agricultural Microbiology, Huazhong Agricultural University, Wuhan, 430070, China; 2 College of Veterinary Medicine, Huazhong Agricultural University, Wuhan, 430070, China; 3 Key Laboratory of Development of Veterinary Diagnostic Products, Ministry of Agriculture, College of Veterinary Medicine, Huazhong Agricultural University, Wuhan, 430070, China; 4 Guangdong Wen’s Group Academy, Guangdong Wen’s Foodstuffs Group Co.,Ltd., Yunfu, 527300, China; 5 Departments of Pathology, College of Veterinary Medicine, University of Georgia, Athens, GA 30602, USA

## Abstract

Virus evolves rapidly to escape vaccine-induced immunity, posing a desperate demand for efficient vaccine development biotechnologies. Here we present an express vaccine development strategy based on CRISPR/Cas9 and Cre/Lox system against re-emerging Pseudorabies virus, which caused the recent devastating swine pseudorabies outbreak in China. By CRISPR/Cas9 system, the virulent genes of the newly isolated strain were simultaneously substituted by marker genes, which were subsequently excised using Cre/Lox system for vaccine safety concern. Notably, single cell FACS technology was applied to further promote virus purification efficiency. The combination of these state-of-art technologies greatly accelerated vaccine development. Finally, vaccination and challenge experiments proved this vaccine candidate’s protective efficacy in pigs and the promise to control current pseudorabies outbreak. This is, to our knowledge, the first successful vaccine development based on gene edit technologies, demonstrating these technologies leap from laboratory to industry. It may pave the way for future express antiviral vaccine development.

Infectious diseases caused by novel emerging and re-emerging viruses pose a continuous threat to our health and economy^1^,^2^,^3^,^4^. Vaccination is the most effective way to prevent viral infectious diseases, which saves millions of lives every year, yet the current long and laborious journey to develop antiviral vaccines is very inefficient^5^. More challengingly, virus evolves rapidly to escape the old vaccine-induced immunity by changing its genome architect^6^. Therefore, new biotechnologies to facilitate and accelerate vaccine development against novel emerging and re-emerging viruses are desperately needed, for the endless arms race with the dynamically evolving virus. Although the genome of DNA viruses is relatively more stable than RNA viruses^7^. There are many studies reported outbreaks of infectious diseases caused by re-emerging DNA virus, such as Adenovirus, Herpes Simplex virus (HSV), Chicken pox virus (Varicella), Hepatitis B virus (HBV), Cytomegalovirus (CMV), etc.^4^,^8^,^9^. Recently, the Pseudorabies virus (PRV), a model herpes virus, was widely prevalent in vaccinated pig farms in China, and caused tremendous economic loss in the swine industry^10^,^11^,^12^,^13^,^14^. PRV is a member of the alpha herpesvirinae subfamily and constitutes approximately 150 K double strand DNA genome^15^. PRV infection caused pseudorabies is one of the most devastating swine infectious diseases in the swine industry worldwide^16^. It has been well controlled for decades by using attenuated and gene deletion vaccines. However, in spite of the great efforts on PRV vaccination, pseudorabies re-emerged as one of the top swine epidemic diseases in recent times, most likely due to PRV mutation caused antigenic drift^10^,^11^. It is conceivable that this phenomenon is occurring in the entire virus community. The laborious and time-consuming traditional vaccine development strategies, including attenuated vaccines and gene deletion vaccines require many rounds of plaque purification or passages and cannot meet the urgent demand for new vaccines. Development of inactivated vaccine is much faster, but requires high dose administration and is generally less effective. Thus, there is an imperative need for novel technologies that could speed up and simplify vaccine development.

Recently, a revolutionary gene-editing technology termed clustered regularly interspaced palindromic repeats (CRISPR)/associated (Cas9) system provided a versatile tool for gene editing^17^,^18^,^19^. With guide RNA (gRNA), Cas9 and its mutant Cas9n protein can specifically break or nick the targeting DNA with high efficiency^17^,^18^,^19^. Subsequently, this will cause indels in the target region by non-homologous end joining (NHEJ) DNA damage repair or foreign genes knocked-in through homologous recombination (HR) in the presence of homologous DNA donor^17^,^18^,^19^. These two pathways are deliberately manipulated for gene editing purposes in different organisms^17^,^18^,^19^,^20^,^21^,^22^,^23^,^24^,^25^. However, the technology of gene editing in DNA viruses is at its infancy. There is evidence that this system can be applied for gene editing in DNA viruses, such as HSV, PRV, Adenovirus and HBV^26^,^27^,^28^. Yet, this technology has neither been demonstrated for simultaneous multi-gene deletion in viral genome nor for successful vaccine development. Cre/Lox system is another high efficient technology extensively used for gene manipulation in many species^29^. Lox sites derived from bacteriophage contains several variants with specific self-compatibility, such as Lox P, Lox N and Lox 2722^29^. All together, application of these modern powerful technologies may potentially accelerate vaccine development.

The aim of the present study is, therefore, to isolate the re-emerging PRV and use it as a model virus to establish a fast and cost-effective technology for express vaccine development.

## Results

### Isolation, characterization and sequence analysis of re-emerging PRV virulent strain

**Figure 1:**
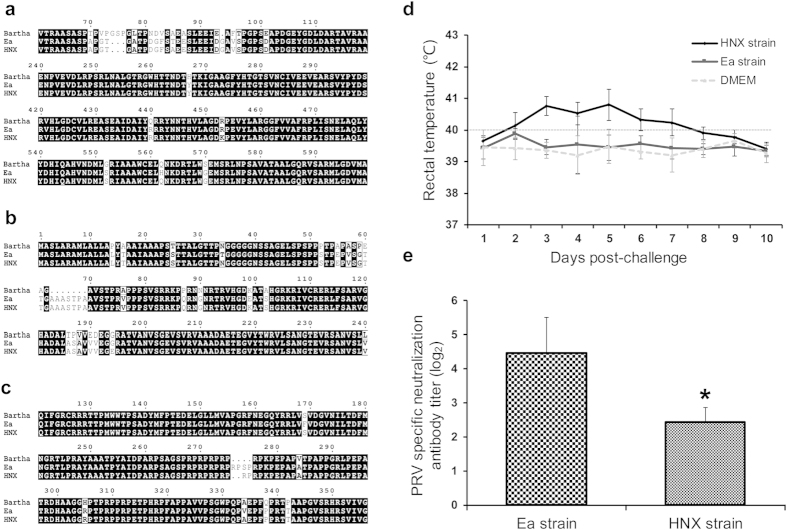
Characterization and sequence analysis of the re-emerging PRV virulent strain. (**a–c**) Amino acid sequence alignments of the main antigen genes gB, gC and gD among PRV HNX strain, PRV Bartha strain and the previous pandemic strain PRV Ea. (**d**) 24 days after vaccination with the commercial PRV vaccine, rectal temperatures of piglets challenged with PRV HNX, Ea strains or DMEM and (**e**) the neutralizing ability of antisera generated against PRV HNX and PRV Ea strain were measured (six piglets per group).

Since 2011, PRV re-emerged and escaped the old vaccine-induced immunity, causing widespread pseudorabies outbreaks in vaccinated pig farms in China^11^. In order to isolate new PRV virulent variants resistant to the current vaccines, PRV-PCR-positive brain samples of aborted fetus from vaccinated pigs were homogenated and inoculated onto PK-15 cells after bacteria filter. A new PRV virulent strain (HNX) was isolated by plaque purification. The major antigens, glycoprotein B (gB), gC and gD of PRV were then amplified by PCR and subjected to sequencing. As shown in Fig. 1a–c, the gB, gC and gD amino acid sequences of PRV HNX displayed some variations and deletion, when compared with the Bartha strain and previous pandemic Ea strain. To further characterize the virulence of PRV HNX strain, PRV-free piglets were intramascularly vaccinated with Bartha-K61, a classical attenuated live vaccine, and then challenged with PRV HNX or Ea strain (10^7^ TCID50). The group challenged with PRV HNX strain developed typical pseudorabies symptoms, whereas no symptoms was observed by the Ea and the uninfected control groups (data not shown). This was further confirmed by the fever (rectal temperature >40.0 °C) in PRV HNX infected group, but not in PRV Ea infected and uninfected control groups (Fig. 1d). Moreover, the virus neutralization titer of antisera was measured 24 days after vaccination. Compared to the neutralization titer of antisera against PRV Ea, the titer against PRV HNX was significantly lower, suggesting the insufficient prophylactic effect of this vaccine against the re-emerging PRV HNX strain (Fig. 1e). Collectively, these data demonstrate that the re-emerging PRV strain can escape the immunity induced by the current commercial vaccines, thus posing an urgent demand for new vaccines.

### PRV multi-virulent genes recombination via CRISPR/Cas9n system

**Figure 2:**
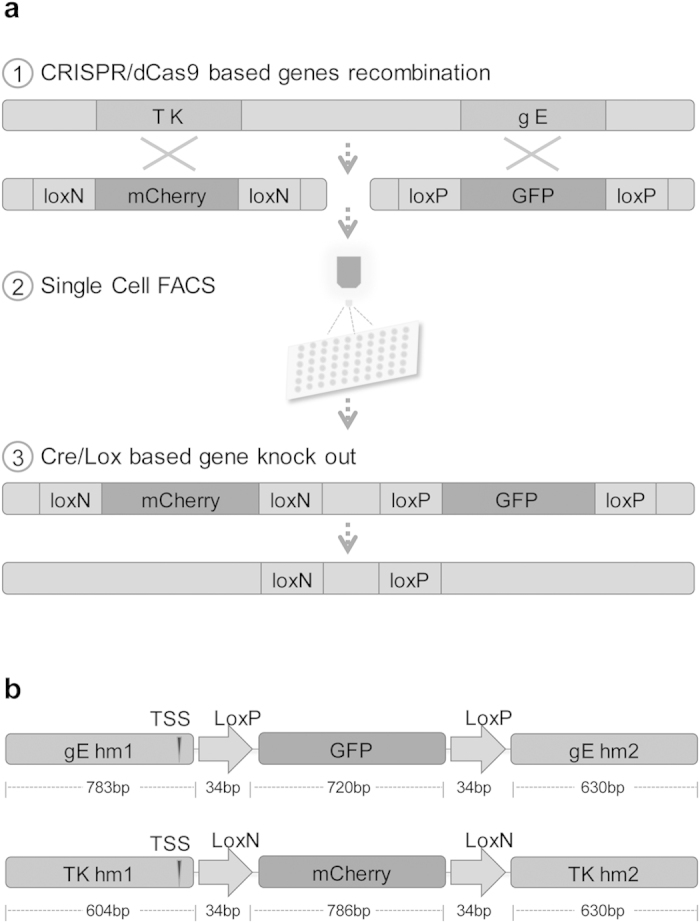
Flowchart of the novel express vaccine development strategy. (**a**) Overview of the strategy for vaccine development. Firstly, PRV virulent gene *gE* and *TK* were simultaneously substituted by GFP and mCherry respectively using CRISPR/Cas9 system assisted homologous recombination. Next, single cell FACS was applied to accelerate recombinant virus purification. Subsequently, GFP and mCherry selection genes were excised by Cre/Lox system. (**b**) Construction of the recombinant DNA donors. mCherry and GFP genes were flanked with LoxP and LoxN sites respectively in same orientation. Transcription start site (TSS) of gE and TK were indicated by arrow.

To establish an express vaccine development strategy, two highly efficient gene edit systems, CRISPR/Cas9 and Cre/Lox system were employed. Meanwhile single cell FACS technique was also applied to simplify and accelerate the plaque purification as shown in Fig. 2a. To avoid false positive signals caused by fluorescent gene expression in homologous recombination donor template, the expressions of GFP and mCherry were driven by endogenous viral promoter, which occurs only after precise DNA homologous recombination. GFP and mCherry genes were flanked with Lox P and Lox N pairs respectively to facilitate fluorescent marker genes excision (Fig. 2b).

**Figure 3:**
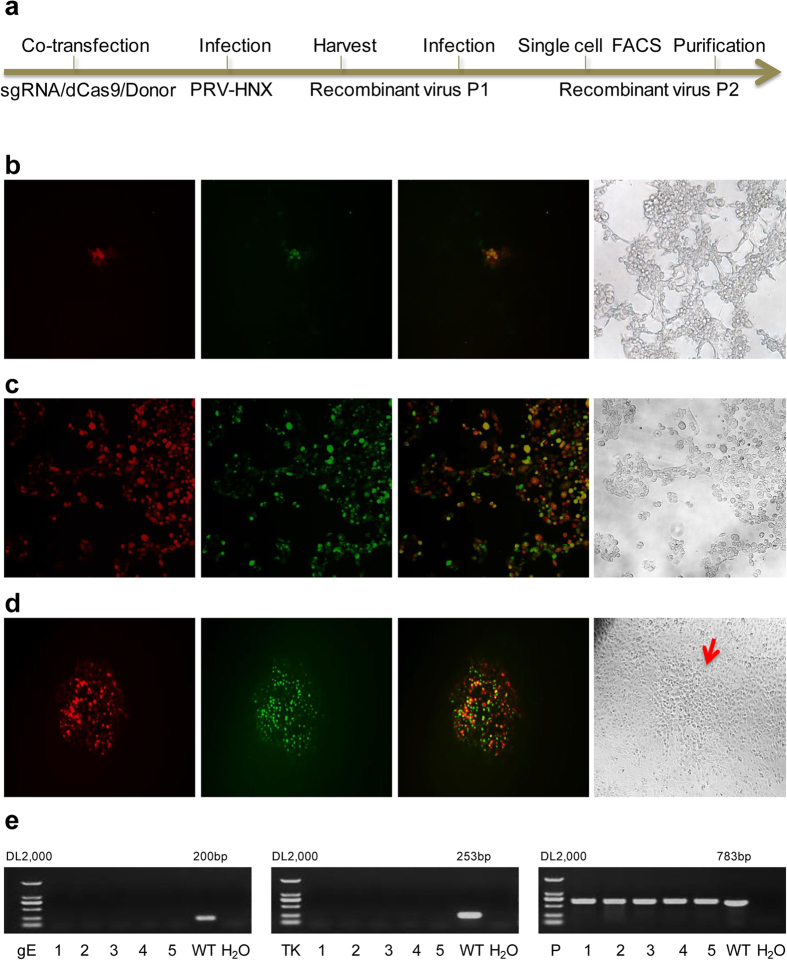
Simultaneous double virulent gene recombination using CRISPR/Cas9 System. (**a**) Procedure of CRISPR/Cas9 system assisted double-gene recombination. (**b**) HEK293T cells co-transfected with sgRNAs and DNA donors and then infected with PRV HNX (MOI = 1). Upon recombination, GFP and mCherry expression will be driven by viral TK and gE promoter respectively. (**c**) Single cells with fluorescent gene recombinant virus were sorted by FACS, plated to 96 well plate pre-cultured with PK15 cells and incubated until fluorescence appeared. (**d**) Plaque purification of double genes recombinant virus in agarose-DMEM plates. Cell plaques were indicated by arrows. (**e**) PCR verification of *TK* and *gE* gene deletion.

Thymidine kinase (*TK*) and *gE* are the major virulent genes of PRV and also the primary targets for PRV gene deletion vaccines^15^. The simultaneous deletion of *TK* and *gE* genes was achieved by CRISPR/Cas9 system mediated homologous recombination. Firstly, sgRNAs target to *TK* and *gE* genes (designed by web based tool http://crispr.mit.edu/) and DNA donors containing fluorescent selection genes and homologous arms were co-transfected to HEK293T cells (Fig. 3a). Eight hours post transfection, cells were then infected with PRV HNX with different MOI (multiplicity of infection) and incubated until recombinant virus expressing red and/or green fluorescence were observed (Fig. 3b). Notably, no green/red overlapping cells were observed in the control group (Data not shown).

Next, the recombinant viruses were collected and inoculated to PK15 cells (P1). PK15 cells with fluorescence were then subjected to FACS and plated one cell per well to 96-well plate pre-cultured with PK15 cells. The wells showing maximum green and red overlapping signals were then subjected one round of plaque purification to obtain the pure recombinant viruses (P2) (Fig. 3c,d). As the viruses with *TK* gene replicate much faster and may thus outgrow the *TK* gene deleted viruses, we applied BVDU (Bromovinyl deoxyuridine), an inhibitor of TK, to suppress WT PRV or *gE* single deletion PRV. Finally, the purity of recombinant virus was validated by PCR amplification. As shown in Fig. 3e, the positive control PCR bands were observed in all the the samples except H_2_O control, indicating the integrity of the viral DNA, whereas *TK* and *gE* genes amplification remained completely negative in PRV recombinant virus.

### Excision of Double fluorescent marker genes via Cre-lox based system

**Figure 4:**
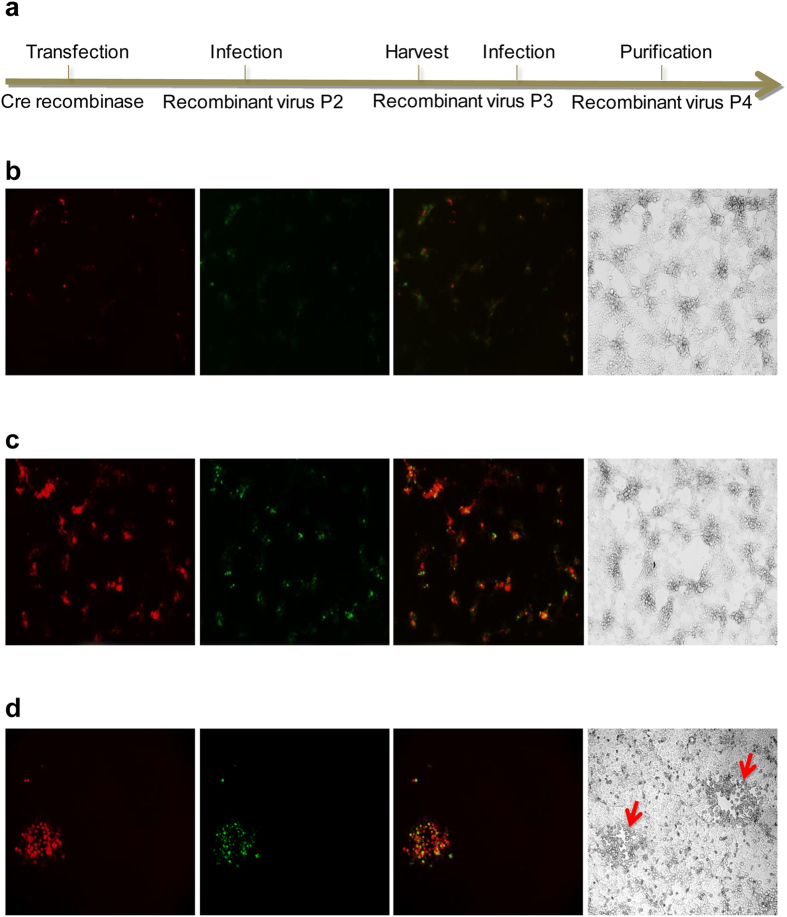
Double fluorescent gene excision using Cre/Lox System. (**a**) Procedure of double fluorescent gene excision by Cre/Lox system. HEK293T cells were transfected with Cre-recombinase (**b**) or control plasmids (**c**) and then infected with fluorescent gene recombinant virus (MOI = 1). The images were taken at 36 hours after infection. (**d**) Plaque purification for double fluorescent genes excision virus. Cell plaques were indicated by arrows.

Due to vaccine safety concerns and regulation, all the selection markers in vaccine candidate need to be completely removed. To this end, the incompatible LoxP and LoxN sites were inserted into the homologous recombination DNA donor, flanking GFP and mChrrey genes respectively in the same orientation (Fig. 2b). HEK293T cells were used for Cre recombinase gene transfection and it mediated gene excision, because of the high transfection and expression efficiency of this cell line. As shown in Fig. 4a, the Cre recombinase gene transfected HEK293T cells were infected with PRV-HNX-TK^−^/gE^−^ GFP^+^ mCherry^+^ recombinant PRV with various MOI (0.1, 1, and 10). After 24 hours of incubation, the Cre recombinase transfected cells exhibit significant less fluorescence compared to the control with mock transfection (Fig. 4b,c). The first generation of Cre-treated recombinant viruses were harvested after infection at an MOI of 1, which showed the highest efficiency for gene excision (P3). Double fluorescent gene excision was achieved through three rounds of plaque purification as shown in Fig. 4d. Thus, by virtue of these genome-editing tools, the re-emerging PRV HNX was successfully engineered into a PRV-HNX-TK^−^/gE^−^ recombinant virus (P4).

### Prophylactic effect of PRV-HNX-TK^−^/gE^−^ in mice and piglets

**Figure 5:**
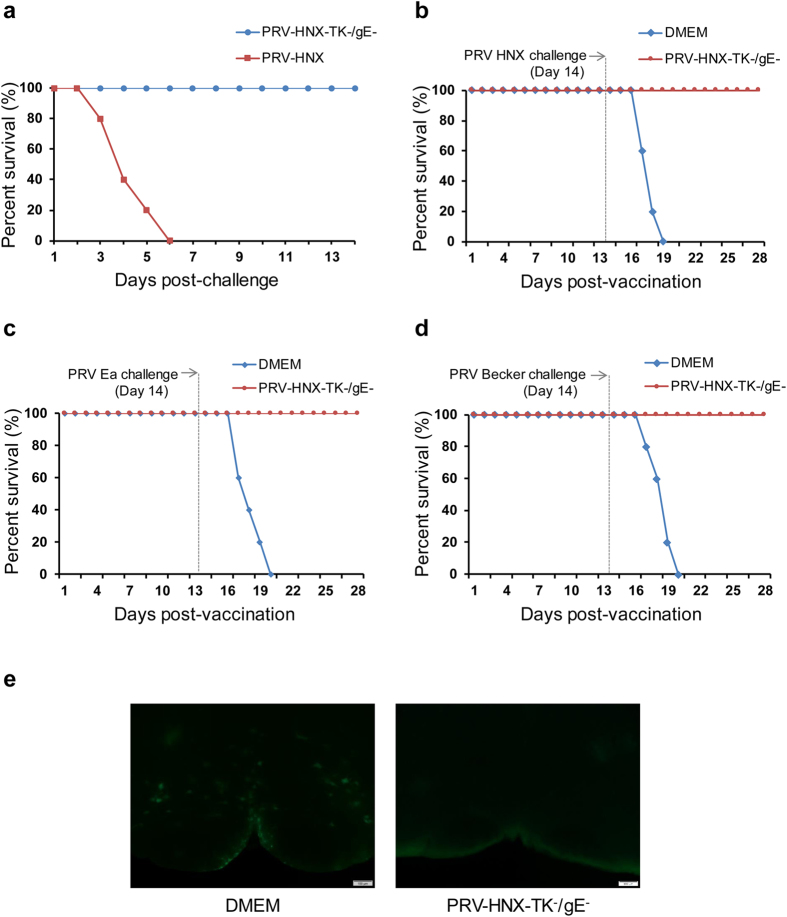
Protective effect of PRV-HNX TK^−^/gE^−^ in mice. (**a**) Two group of mice were subcutaneously injected with PRV HNX or PRV-HNX TK^−^/gE^−^ and were monitored for survival rate for two weeks. Mice were vaccinated PRV-HNX TK^−^/gE^−^ or DMEM and then challenged with PRV HNX (**b**), PRV Ea (**c**) and PRV-Becker-GFP (**d**) two weeks later. Survival rate were monitored for two weeks. (**e**) Fluorescence imaging of the brain stem section of the mice vaccinated PRV-HNX TK^−^/gE^−^ or DMEM respectively and then challenged with PRV-Becker-GFP (five mice per group).

The safety of this novel vaccine candidate was firstly assessed in mice. All mice injected with PRV HNX group died, three to five days later, whereas all the PRV-HNX-TK^−^/gE^−^ injected mice remained healthy (Fig. 5a). Next, the PRV-HNX-TK^−^/gE^−^ vaccinated mice were challenged with the re-emerging PRV HNX, PRV Ea or a standard WT virus strain PRV-Becker with GFP in gE position. Fig. 5b–d demonstrated that vaccination with PRV-HNX-TK^−^/gE^−^ provides complete protection against the previous and re-emerging PRV strains. This was further supported by the presence of extensive GFP signals in brain sections of unvaccinated mice but not in that of the vaccinated group (Fig. 5e).

**Figure 6:**
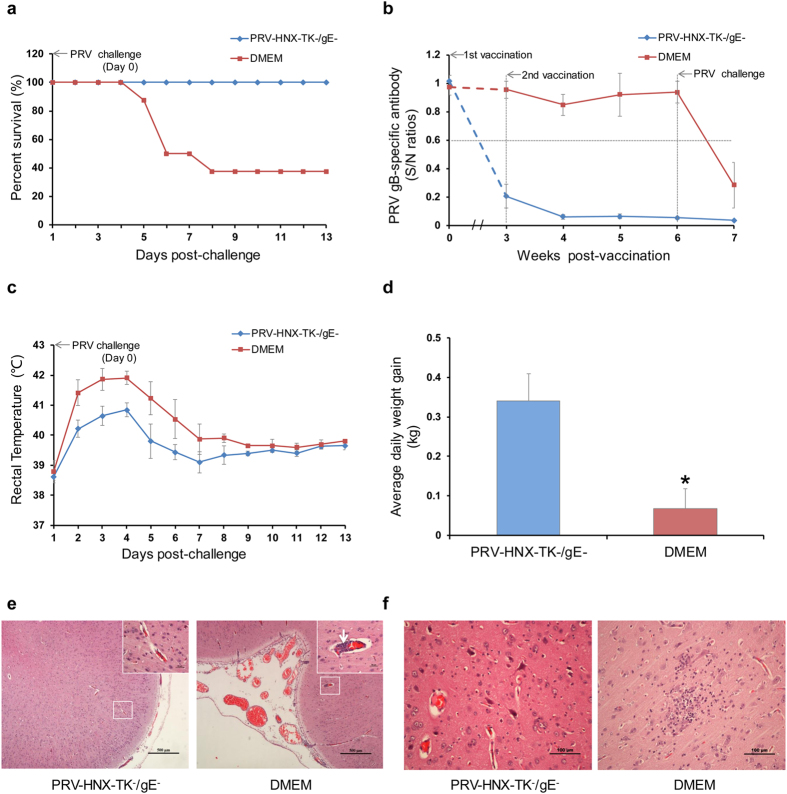
Protective effect of PRV-HNX TK^−^/gE^−^ in piglets. Two groups of piglets were vaccinated with PRV-HNX TK^−^/gE^−^ or DMEM respectively, and then challenged with PRV HNX. (**a**) Survival rate, (**b**) gB-specific antibody ELISA, (**c**) body temperature, and (**d**) average daily weight gain were recorded. (**e,f**) Histological imaging of the brain section of the piglets vaccinated PRV-HNX TK^−^/gE^−^ or DMEM respectively, and then challenged with PRV HNX. (≤0.6 = positive, 0.6–0.7 = suspect, >0.7 = negative).

Next, sixteen of PRV-free piglets were randomly divided into two groups (one group immunized with PRV-HNX-TK^−^/gE^−^ and the other with DMEM) and subsequently challenged with current pandemic PRV (10^8^ TCID50) intranasally at 28 days post vaccination. Death, anti-gB antibodies, and clinical signs including body temperature, respiratory distress, depression, anorexia, cough, convulsions, ataxia, itching, and weight were measured or recorded. No typical pseudorabies clinical signs were observed in PRV-HNX-TK^−^/gE^−^ vaccinated group, whereas severe pseudorabies symptoms were developed in the control group (data not shown). As shown in Fig. 6a, this vaccine candidate can completely protect piglets against the current pandemic PRV strain, while only two piglets survived in the control group. In this line, anti-gB antibody was significantly increased post vaccination in vaccinated piglets (Fig. 6b). The fever (rectal temperature >40.0 °C) and daily weight gain of vaccinated group were significantly more than that of the control group (Fig. 6c,d). Next, the dead and surviving piglets were subjected to histopathological analysis. Piglets from the control group exhibited severe pathological damages in brain, such as obvious meningeal inflammation, microglia nodule and perivascular cuff, whereas just a mild meningeal inflammation were observed in the vaccinated piglets even after challenge with high dose of virus (10^8^ TCID50) (Fig. 6e,f). Together, these data demonstrated the potential of this vaccine candidate for protection against pseudorabies.

## Discussion

Despite of the interdisciplinary efforts, it is still time-consuming and laborious to develop vaccines against the constantly changing viruses^5^. In this study, we isolated a re-emerging PRV and used it as a model to establish a fast and cost-effective technology for express vaccine development.

The traditional way to develop multiple gene deletion vaccine involves several steps of single gene recombination and marker gene excision. To obtain a PRV double gene deletion vaccine candidate, it requires about twenty rounds of time-consuming plaque purification^5^. We combined two highly efficient gene edit systems, CRISPR/Cas9 system and Cre/Lox system to increase the multi-gene editing efficiency in viral genome. Meanwhile, single cell FACS technology was also employed to further promote virus purification efficiency. We can indeed obtain PRV-HNX-TK^−^/gE^−^/GFP^+^/mCherry^+^ recombinant PRV by just a single round of plaque purification, whereas it requires about ten rounds of plaque purification by traditional strategy. Moreover, owning to the high efficiency of Cre recombinase and incompatible LoxP and LoxN sites, the multiple marker genes excision can be achieved simultaneously in one step followed by just three rounds of plaque purification. Of note, in our study, the expression of selection fluorescent genes was driven by endogenous viral promoter, which occurs only after precise DNA homologous recombination. In this scenario, we could avoid the false positive due to basal fluorescent gene expression and can thus utilize single cell sorting as an update for traditional time-consuming viral plaque purification. Together, this strategy greatly facilitates and accelerates PRV vaccine development to simply one step of simultaneous multiple-gene recombination mediated by CRISPR/Cas9 system, and one step of simultaneous multiple marker genes excision mediated by Cre/Lox system. It requires just only four rounds of plaque purification in total to obtain a double gene deletion vaccine candidate.

This strategy could also be applied to vaccine development against other DNA virus such as HSV, CMV, Adenovirus, VZV, Duck Hepatitis Virus (DHV), Bovine Herpesvirus (BHV) and Epstein Barr Virus (EBV). Yet the CRISPR/Cas9 system is mostly applied in DNA editing, accumulating data suggest that this versatile technology could also work effectively in the RNA world. O’Connell *et al*. demonstrated that trans-complementation of protospacer adjacent motif presenting oligonucleotides (PAMmers) can indeed stimulate site-specific targeting and cleavage of ssRNA by Cas9 endonuclease^30^. Recently, *Francisella novicida* Cas9 has been shown to have the capability of targeting endogenous bacterial RNA and was engineered to target and inhibit hepatitis C virus, a ssRNA virus^31^. It is conceivable that with the advances in this dynamic research field, CRISPR/Cas system could be very possibly employed to develop anti-RNA virus vaccines. Moreover, as recombinant PRV and HSV are extensively exploited for neural circuit tracing, our strategy could also significantly simplify the recombinant viral tracer engineering and could thus contribute to neuroscience research as well^32^.

While it has been shown that the CRISPR system can be used in viral genome editing^26^,^27^, the application of this technology in viruses is at its infancy. CRISPR-based simultaneous multiple viral genes editing and vaccine development has never been reported. Here, we simultaneously replaced two main virulent PRV genes, *gE* and *TK*, by GFP and mCherry respectively using CRISPR/Cas9 system assisted homologous recombination. In contrary to a recent study which showed the CRISPR/Cas9 system could only work with extracted PRV viral genome DNA^27^, our data proved this system is also able to directly edit PRV genome during viral infection. In this line, it has been reported that CRISPR system introduced into host cells during Adenovirus and HSV replication can indeed robustly stimulate DNA breakages in the targeted genomes with high frequency^26^. This discrepancy could be possibly due to the application of *TK* gene inhibitors in our experiments to prevent WT viruses outgrow the gene deletion viruses.

Generally, the genome of DNA viruses is relatively more stable than that of RNA viruses^7^, however, there are many studies reporting outbreaks of infectious diseases caused by newly or re-emerging DNA viruses^4^,^8^,^9^. Here, we isolated a re-emerging, PRV HNX resistant to the classical PRV vaccines. The sequence analysis revealed several mutations/deletion in gB, gC and gD of PRV. It would be of great interesting to further investigate whether these mutations/deletions could cause the antigenic drift, and if so, what is the molecular mechanism leading to the resistance to the vaccine induced immunity in the swine population^10^,^11^,^14^. Here, our animal experiments in mouse and piglets validated the protective effect of this gene editing technologies based new vaccine candidate, demonstrating its potential in controlling the current pseudorabies outbreak in the swine industry.

Together, we combined several state-of-art modern biotechnologies to establish a novel strategy for express vaccine development and obtained, to our knowledge, the first successful gene editing technologies based vaccine candidate. This new generation biotechnologies based strategy might open an express avenue for future anti-viral vaccine development.

## Materials and Methods

### Plasmid construction, viral genomic preparation and PCR amplification

The guide RNAs were cloned into sgRNA/Cas9n expression vector (Addgene plasmid px335) as previously described^17^. gE and TK homologous arms were amplified from PRV HNX strain by PCR. gEhm1-loxP-GFP-loxP-gEhm2 and TKhm1-loxN-mCherry-loxN-TKhm2 donor templates were constructed using overlapping PCR. The GFP were inserted into gE position in PRV-Becker BAC (a kind gift from Dr. L. W. Enquist) of which the virus were rescued in PK15 cell as previously described^33^. The PRV genomic DNA was extracted using the TIANamp virus DNA kit (TIANGEN) following the manufacturer’s protocol. All the sequence of primers, sgRNAs, donor templates and antigenic genes are listed in supplementary data.

### Cell culture, DNA transfection and viral infection

HEK293T cells and PK15 cells were cultured in DMEM with 10% FBS, and streptomycin/penicillin. DNA was transfected into HEK293T and PK15 cells using Lipofectamine. PRV infection was performed as previously described^26^. The culture medium was changed to DMEM with 2% FBS after transfection and viral infection.

### DNA synthesis, sequencing and analysis

The PCR primer synthesis and DNA sequencing were done by Shanghai Sangon Biotch, China. gB, gC, and gD gene sequences of PRV Ea strain obtained from NCBI were analyzed by BioEdit software.

### Virus preparation, propagation and plaque purification

Virus infected PK15 cells were harvested at 24 hours post-infection. After three rounds of freeze-thaw, the cell lysate was centrifuged for 5 minutes at 3,000 rpm/min. The supernatant were used for next propagation or stored at −80 °C. For virus purification, the infected PK15 cells were covered by mixture with 2 × DMEM and 1.6% low melting-point agarose (20 μg ml^−1^ BVDU when necessary). After 36–48 hours, well-separated plaques were picked up by pipette tip and was pipetted into micro-tubes containing 200 μl serum free DMEM for next propagation or plaque purification.

### Fluorescence-activated cell sorting

The fluorescence-activated cell sorting was performed on MoFlo XDP sorter (Beckman Coulter) following the manufacturer’s protocol. PK15 cells were infected with the first generation recombinant PRV at an MOI of 0.01. The single cells expressing fluorescent proteins were sorted into 96-well plate pre-seeded with 5,000 PK15 cells.

### Animal experiments

Female BALB/C mice were immunized with 10^4.7^ TCID50 PRV-HNX TK^−^/gE^−^ and inoculated subcutaneously with PRV HNX, PRV Ea or PRV Becker two weeks later. For piglet experiments, 10^5^ TCID50 PRV-HNX TK^−^/gE^−^ or PRV Bartha-K61 injected intramuscularly for immunization, booster vaccination was performed 14 days later. The piglets were intranasally challenged with PRV at 28 days post second immunization. Clinical signs were recorded daily for up to 14 days. All of the animal experiments were approved by the Research Ethics Committee, Huazhong Agricultural University, Hubei, China (HZAUMO2015-0015). All the animal experiments were carried out in accordance with the recommendations in the Guide for the Care and Use of Laboratory Animals from Research Ethics Committee, Huazhong Agricultural University, Hubei, China.

### ELISA, neutralization assay and histopathology

The serum samples of PRV-specific gB antibodies were evaluated using commercial ELISA kits according to the manufacturer’s directions (IDEXX, USA). The neutralizing antibody against PRV was tested as described previously^34^. The brain tissues were fixed with 4% paraformaldehyde solution at room temperature for 2 days and then processed by routine histopathological procedures as previously described^35^.

### Statistical analysis

The significant differences of the animal experiments were analyzed using two-way ANOVA or/and t-test in the GraphPad Prism (version 5.01) software (San Diego, CA). Differences were considered statistically significant when P < 0.05.

## Additional Information

**How to cite this article**: Liang, X. *et al*. A CRISPR/Cas9 and Cre/Lox system-based express vaccine development strategy against re-emerging pseudorabies virus. *Sci. Rep*. **6**, 19176; doi: 10.1038/srep19176 (2016).

## Supplementary Material

Supplementary Information
