# Supplementary material for: A CRISPR/Cas9 and Cre/Lox system-based express vaccine development strategy against re-emerging Pseudorabies virus
Source: Sci Rep. 2016 Jan 18;6:19176. doi: 10.1038/srep19176 (PMC4726036; doi:10.1038/srep19176)
Supplement: Supplementary Information [file srep19176-s1.pdf]

A CRISPR/Cas9 and Cre/Lox system-based express vaccine development strategy against re-emerging Pseudorabies virus

Xun Liang[1,2+], Leqiang Sun[1,2+], Teng Yu[1,2+,] , Yongfei Pan[4], Dongdong,Wang[4], Xueying Hu[2], Zhenfang Fu[5], Qigai He[1,2,3*], Gang Cao[1,2,3*]

[1]State Key Laboratory of Agricultural Microbiology, Huazhong Agricultural University, Wuhan, 430070, China.

[2]College of Veterinary Medicine, Huazhong Agricultural University, Wuhan, 430070, China.

[3]Key Laboratory of Development of Veterinary Diagnostic Products, Ministry of Agriculture, College of Veterinary Medicine, Huazhong Agricultural University, Wuhan 430070

[4]Guangdong Wen's Group Academy, Guangdong Wen's Foodstuffs Group Co.,Ltd., Xinxing, Guangdong, China.

[5]Departments of Pathology, College of Veterinary Medicine, University of Georgia, Athens, GA 30602, USA.

[+]These authors contributed equally to this work

*Address correspondence to:

Qigai He, State Key Laboratory of Agricultural Microbiology NO.1 Shizishan, Nanhu, Wuhan, 430070, China

Email: he628@mail.hzau.edu.cn

Gang Cao, State Key Laboratory of Agricultural Microbiology NO.1 Shizishan, Nanhu, Wuhan, 430070, China

Email: gcao@mail.hzau.edu.cn

Supplementary Data

FigS1. Sequences of PRV HNX gB, gC and gD genes.

PRV HNX-gB

ATGCCCGCTGGTGGCGGTCTTTGGCGCGGGCCCCGCGGGCATCGGCCCGGGCACCACGGCGGTGCTGGCCTCGGACGTCTTTGGCCTG

CTCCACACCACGCTGCAGCTGCGCGGGGCGCCGTCGCGCTAGCGCTGCTGCTGCTGGCGCTCGCCGCGACCCCGACGTGCGGCGCGGC

GGCCGTGACGCGGGCCGCCTCGGCCTCGCCCGCGCCCGGGACGGGCGCCACCCCAGACGGCTTCTCCGCGGAGGAGTCCCTCGAGGA

GATCGACGGGGCCGTCTCCCCCGGCCCCTCGGACGCCCCCGACGGCGAGTACGGCGACCTGGACGCGCGCACGGCCGTGCGCGCGGC

CGCGACCGAGCGGGACCGCTTCTACGTCTGCCCGCCGCCGTCCGGCTCCACGGTGGTGCGCCTGGAGCCCGAGCAGGCCTGCCCCGAG

TACTCGCAGGGGCGCAACTTCACGGAGGGGATCGCCGTGCTCTTCAAGGAGAACATCGCCCCGCACAAGTTCAAGGCCCACATCTACTACA

AGAACGTCATCGTCACGACCGTGTGGTCCGGGAGCACGTACGCGGCCATCACGAACCGCTTCACGGACCGCGTGCCCGTCCCCGTGCAG

GAGATCACGGACGTGATCGACCGCCGCGGCAAGTGCGTCTCCAAGGCCGAGTACGTGCGCAACAACCACAAGGTGACCGCCTTCGACCGC

GACGAGAACCCCGTCGAGGTGGACCTGCGCCCCTCGCGCCTGAACGCGCTCGGCACCCGCGGCTGGCACACCACCAACGACACCTACAC

CAAGATCGGCGCCGCGGGCTTCTACCACACGGGCACCTCCGTCAACTGCATCGTCGAGGAGGTGGAGGCGCGCTCCGTGTACCCCTACGA

CTCCTTCGCCCTGTCCACGGGGGACATCGTGTACATGTCCCCCTTCTACGGCCTGCGCGAGGGGGCCCACGGGGAGCACATCGGCTACGC

GCCCGGGCGCTTCCAGCAGGTGGAGCACTACTACCCCATCGACCTGGACTCGCGCCTCCGCGCCTCCGAGAGCGTGACGCGCAACTTTCT

GCGCACGCCGCACTTCACGGTGGCCTGGGACTGGGCCCCCAAGACGCGGCGCGTGTGCAGCCTGGCCAAGTGGCGCGAGGCCGAGGAG

ATGATCCGCGACGAGACGCGCGACGGGTCCTTCCGCTTCACGTCGCGGGCCCTGGGCGCCTCCTTCGTCAGCGACGTCACGCAGCTCGA

CCTGCAGCGCGTGCACCTGGGCGACTGCGTCCTCCGCGAGGCCTCGGAGGCCATCGACGCCATCTACCGGCGGCGCTACAACAACACGC

ACGTGCTGGCCGGCGACAAGCCCGAGGTGTACCTCGCCCGCGGGGGCTTCGTGGTGGCCTTCCGCCCGCTGATCTCGAACGAGCTGGCG

CAGCTGTACGCGCGCGAGCTCGAGCGCCTCGGCCTCGCCGGCGTCGTGGGCCCCGCGTCCCCCGCGGCCGCCCGTCGGGCCCGGCGC

TCCCCCGGCCCGGCGGGGACGCCCGAGCCGCCGGCCGTCAACGGCACGGGGCACCTGCGCATCACCACGGGCTCGGCCGAGTTTGCGC

GCCTGCAGTTCACCTACGACCACATCCAGGCGCACGTGAACGACATGCTGAGCCGCATCGCGGCCGCCTGGTGCGAGCTGCAGAACAAGG

ACCGCACCCTGTGGGGCGAGATGTCGCGCCTGAACCCCAGCGCCGTGGCCACGGCCGCGCTGGGCCAGCGCGTCTCGGCGCGCATGCT

CGGCGACGTGATGGCCATCTCGCGGTGCGTGGAGGTGCGCGGCGGCGTGTACGTGCAGAACTCCATGCGCGTGCCCGGCGAGCGCGGC

ACGTGCTACAGCCGCCCGCTGGTGACCTTCGAGCACAACGGCACGGGCGTGATCGAGGGCCAGCTCGGCGACGACAACGAGCTCCTCAT

CTCGCGCGACCTCATCGAGCCCTGCACCGGCAACCACCGGCGCTACTTTAAGCTGGGCGGCGGGTACGTGTACTACGAGGACTACAGCTA

CGTGCGCATGGTGGAGGTGCCCGAGACGATCAGCACGCGGGTGACCCTGAACCTGACGCTGCTCGAGGACCGCGAGTTCCTGCCCCTCG

AGGTGTACACGCGCGAGGAGCTCGCCGACACGGGCCTCCTGGACTACAGCGAGATCCAGCGCCGCAACCAGCTGCACGCGCTCAAGTTCT

ACGACATTGACCGCGTGGTCAAGGTGGACCACAACGTGGTGCTGCTGCGCGGCATCGCCAACTTCTTCCAGGGCCTCGGCGACGTGGGC

GCCGCCGTCGGCAAGGTGGTCCTGGGCGCCACGGGGGCCGTGATCTCGGCCGTCGGCGGCATGGTGTCCTTCCTGTCCAACCCCTTCGG

GGCGCTCGCCATCGGGCTGCTGGTGCTGGCCGGCCTGGTCGCGGCCTTCCTGGCCTACCGGCACATCTCGCGCCTGCGCCGCAACCCCA

TGAAGGCCCTGTACCCCGTCACGACGAAGGCGCTCAAGGAGGACGGCGTCGAAGAGGACGACGTGGACGAGGCCAAGCTGGACCAGGC

CCGGGACATGATCCGGTACATGTCCATCGTGTCGGCCCTCGAGCAGCAGGAGCACAAGGCGCGCAAGAAGAACAGCGGGCCCGCGCTGC

TGGCCAGCCGCGTCGGGGCGATGGCCACGCGCCGCCGGCACTACCAGCGCCTCGAGAACGAGGACCCCGACGCCCCCTAG

PRV HNX-gC

ATGGCCTCGCTCGCGCGTGCGATGCTCGCGCTGCTGGCGCTCTACACGGCGGCCATCGCCGCGGCGCCGTCGTCCACGACGGCGCTCGG

CACGACGCCCAACGGGGGCGGGGGCGGCAACAGCAGCGCGGGCGAGCTCTCGCCCTCGCCGCCCTCGACGCCCGAGCCCGTCTCGGG

GACGACGGGGGCCGCGGCCTCCACGCCCGCCGCCGTCTCGACGCCCCGGGTCCCGCCGCCCTCGGTCTCGCGCCGGAAGCCCCAGCG

GAACGGCAACAGGACGCGCGTCCACGGCGACAAGGCCACCTCGCACGGGCGCAAGCGCATCGTGTGCCGCGAGCGGCTGTTCTCGGCG

AGGGTGGGGGACGCGGTCAGCTTCGGGTGCGCCGTCGTCCCGCGCGCCGGGGAGACCTTCGAGGTCCGCTTCTGCCGCCGCGGGCGCT

TCCGCTCGCCCGACGCCGACCCCGAGTACTTTGACGAGCCCCCGCGCCCGGAGCTCCCGCGGGAGCGGCTCCTCTTCAGCTCCGCCAAC
GCCTCCCTCGCCCACGCGGACGCGCTCGCCTCCGCCGTCGTCGTCGAGGGCGAGCGCGCGACCGTCGCCAACGTCTCGGGCGAGGTGT
CCGTGCGCGTGGCCGCGGCGGACGCCGAGACCGAGGGCGTCTACACGTGGCGCGTGCTGTCCGCCAACGGCACCGAGGTCCGCAGCGC
CAACGTCTCGCTCGTCCTGTACCACCAGCCCGAGTTCGGCCTGAGCGCGCCGCCCGTCCTCTTCGGCGAGCCCTTCCGGGCGGTGTGCG
TCGTCCGCGACTACTACCCGCGGCGCAGCGTGCGCCTGCGCTGGTTCGCGGACGAGCACCCGGTGGACGCCGCCTTCGTGACCAACAGC
ACCGTGGCCGACGAGCTCGGGCGCCGCACGCGCGTCTCCGTGGTGAACGTGACGCGCGCGGACGTCCCGGGCCTCGCGGCCGCGGAC
GACGCGGACGCGCTCGCGCCGAGCCTGCGCTGCGAGGCCGTGTGGTACCGCGACAGCGTGGCCTCGCAGCGCTTCTCCGAGGCCCTGC
GCCCCCACGTCTACCACCCGGCGGCGGTCTCGGTGCGCTTCGTCGAGGGCTTCGCCGTCTGCGACGGCCTCTGCGTGCCCCCGGAGGC
GCGCCTCGCCTGGTCCGACCACGCCGCCGACACCGTCTACCACCTCGGCGCCTGCGCCGAGCACCCCGGCCTGCTCAACGTGCGGAGC
GCCCGCCCGCTGTCGGACCTCGACGGGCCCGTCGACTACACCTGCCGCCTCGAGGGCATGCCCTCGCAGCTGCCCATCTTCGAGGACAC
GCAGCGCTACGACGCCTCCCCCACGTCCGTGAGCTGGCCCGTCGTGACCAGCATGATCACCGTCATCGCCGGCATCGCCATCCTAGCCATC
GTGCTGGTCATCATGGCGACGTGCGTCTACTACCGCCGGTCCGCGCTGTGA

PRV HNX-gD

ATGCTGCTCGCAGCGCTATTGGCGGCGCTGGTCGCCCGGACGACGCTCGGCGCGGACGTGGACGCCGTGCCCGCGCCGACCTTCCCCCC
GCCCGCGTACCCGTACACCGAGTCGTGGCAGCTGACGCTGACGACGGTCCCCTCGCCCTTCGTCGGCCCCGCGGACGTCTACCACACGC
GCCCGCTGGAGGACCCGTGCGGGGTGGTGGCGCTGATCTCCGACCCGCAGGTGGACCGGCTGCTGAACGAGGCGGTGGCCCACCGGCG
GCCCACGTACCGCGCCCACGTGGCCTGGTACCGCATCGCGGACGGGTGCGCGCACCTGCTGTACTTTATCGAGTACGCCGACTGCGACCC
CAGGCAGATCTTTGGGCGCTGCCGGCGCCGCACCACGCCGATGTGGTGGACCCCGTCCGCGGACTACATGTTCCCCACGGAGGACGAGC
TGGGGCTGCTCATGGTGGCCCCGGGGCGGTTCAACGAGGGCCAGTACCGGCGCCTGGTGTCCGTCGACGGCGTGAACATCCTCACCGAC
TTCATGGTGGCGCTCCCCGAGGGGCAAGAGTGCCCGTTCGCCCGCGTGGACCAGCACCGCACGTACAAGTTCGGCGCGTGCTGGAGCGA
CGACAGCTTCAAGCGGGGCGTGGACGTGATGCGATTCCTGACGCCGTTCTACCAGCAGCCCCCGCACCGGGAGGTGGTGAACTACTGGTA
CCGCAAGAACGGCCGGACGCTCCCGCGGGCCTACGCCGCCGCCACGCCGTACGCCATCGACCCCGCGCGGCCCTCGGCGGGCTCGCCG
AGGCCCAGGCCCCGGCCCCGGCCCAGGCCCCGGCCGAAGCCCGAGCCCGCCCCGGCGACGCCCGCGCCCCCGGCCGCCTGCCCGAG
CCGGCGACGCGGGACCACGCCGCCGGGGGGCGCCCCACGCCGCGACCCCCGAGGCCCGAGACGCCGCACCGCCCCTTCGCCCCGCCG
GCCGTCGTGCCCAGCGGGTGGCCGCAGCCCGCGGAGCCGTTCCCGCCCCGGACCACCGCCGCGCCGGGCGTCTCGCGCCACCGCTCG
GTGATCGTCGGCACGGGCACCGCGATGGGCGCGCTCCTGGTGGGCGTGTGCGTCTACATCTTCTTCCGCCTGAGGGGGGCGAAGGGGTA
TCGCCTCCTGGGCGGTCCCGCGGACGCCGACGAGCTAAAAGCGCAGCCCGGTCCGTAG

FigS2. Sequence of PRV gE gene recombination donor template (gEhm1-loxP-GFP-loxP-gEhm2). gE hm1 and gE hm2 colored in green, loxP sites colored in yellow, GFP colored in blue, the endogenous TSS of PRV gE shaded in violet.

gaacgggaccacggacctctttgtgctgacggccctggtgccgccgagggggcgccccgtccccacgtcgccgcccgcggacgagtgccggc
ccgtcgtcggatcgtggcacgacagcctgcgcgtcgtggaccccgccgaggacgccgtgttcaccacccagcccccgcccgagcccgagccg
ccgacgacccccgcgccccccgggggaccggcgccacccccgagccccgatcggacgaggaggaggagggtgacgcggagacgacg
acgccgacgctgaccccggcgcccgggaccctggacgcgaacggcacgatggtgctgaacgccagcgtcgtgtcgcgcgtcctgctcgccgc
cgccaacgccacggcgggcgcccggagccccgggaagatagccatggtgctggggcccacgatcgtcgtcctcctgatcttcctgggcgggat
cgcctgcgtggcccggcgctgcgcgcggaatcgcatctaccggccgcgacccgggcgcggatcggcggtccatgcggcgcccccgcggcgc
ccgccccccaaccccgtcgccggggcgcccgtcccccagcccaagatgacgttggccgagctgcgccagaagctcgccaccatcgcagaag

aacaataaaaaggtggtgtttgcataattttgtgggtggcgttttatctccgtccgcgccgttttaaacctgggcacccccgcgagtctcgcacacacc
ggggttgagaccatgcggccctttctgctgcgcgccgcgcagataccggggcggcaggccctgccaataacttcgtatagcatacattatacgaa
gttatatcgattgaattcgggaggaggaggatccgtgagcaagggcgaggagctgttcaccggggtggtgcccatcctggtcgagctggacggc
gacgtaaacggccacaagttcagcgtgtccggcgagggcgagggcgatgccacctacggcaagctgaccctgaagttcatctgcaccaccgg
caagctgcccgtgccctggcccaccctcgtgaccaccttcggctacggcctgcagtgcttcgcccgctaccccgaccacatgaagcagcacgac
ttcttcaagtccgccatgcccgaaggctacgtccaggagcgcaccatcttcttcaaggacgacggcaactacaagacccgcgccgaggtgaagt
tcgagggcgacaccctggtgaaccgcatcgagctgaagggcatcgacttcaaggaggacggcaacatcctggggcacaagctggagtacaa
ctacaacagccacaacgtctatatcatggccgacaagcagaagaacggcatcaaggtgaacttcaagatccgccacaacatcgaggacggc
agcgtgcagctcgccgaccactaccagcagaacacccccatcggcgacggccccgtgctgctgcccgacaaccactacctgagctaccagtc
cgccctgagcaaagaccccaacgagaagcgcgatcacatggtcctgctggagttcgtgaccgccgccgggatcactctcggcatggacgagct
gtacaagggaggaggaggatccgaagcttgcctcgagcagcgctgctcgagagatctataacttcgtatagcatacattatacgaagttatataac
ttcgtatagcatacattatacgaagttataaccgcctgttgatgtcccgccccgcttaaataccgggagaaccggtccgcccgcattccgacatgcc
cggcgccgcctccgtcgacatggacacgtttgaccccagcgcccccgtcccgacgagcgtctcgaacccggccgccgacgtcctgctggcccc
caagggaccccgctccccgctgcgcccccaggacgactcggactgctactacagcgagagcgacaacgagacgcccagcgagttcctgcgc
cgcgtgggacgccggcaggcggcgcgtcggagacgccgccgctgcctgatgggcgtcgcgatcagcgccgccgcgctggtcatctgctcgct
gtccgcgctactcgggggcatcgtcgccaggcacgtgtagcgagcgagcgagcgaacgggagcgggggcccgcccccatccgccgcgccc
aggagagggggggagagagcgggggggttgggcgcgccacgtggtgtgggcacggactcggacttgtcacaataaatgggccccggcgtgtcc
gggcgcacacagcagccttcctctcctccgcgtctctgttccgcccgtctctcgccggactcttcttctccaccgcctccaccgtcgcagttgtcgcga
gcgcgt

FigS3 Sequence of PRV TK gene recombination donor template (TKhm1-loxN-mCherry-loxN-TKhm2). TKhm1 and TKhm2 colored in green, loxN sites colored in grey, mCherry colored in red, the endogenous TSS of PRV TK shaded in violet.

ccgccttatcatccccgctccccgccgccgcccggcccggccccgcgcgcgccgcgatcgcgatcaccgccgcggcccggcgacgtactcgg
cgaggccgcgcacggtcgcggccatcgcgctcgcgttgccgcgcgtctgggtgcagggcaggcgcgtcacgtcgagcacgcgcatgctccgct
gggccacaaacaccagcaggggcacgagcgtgatctcctcgccgcccgggggcacggcggcggcgaggaggcgcgccgagtcgcgcag
ctggcacagcccctcgtgccgctgcccgcgcttgctgggcgtgttgaggttccgggggaagcggcacgtcttgagctcgatgaggaagcacagg
tgcgggcccgcccccagccgcaccacgcacacgcagtcggggcggcgcaccccgaggttgacttcaaaggccagggtcaaggacgccttctt
aagcgtctctcggggaagcccgaagagactctcgccgtacgcggacgggtcgcgtcgcaggcgttcgtagaagcggttgtggcagcggatccc
cgcccggaagcgcgccgggatgcgcatcctccggatctacctcgacggcgcccttccatttcaggtgtcgtcaggtacctctagaataacttcgtat
agtataccttatacgaagttatccggggtggatcctggcggcggctccgtgagcaagggcgaggaggataacatggccatcatcaaggagttcat
gcgcttcaaggtgcacatggagggctccgtgaacggccacgagttcgagatcgagggcgagggcgagggccgcccctacgagggcaccca
gaccgccaagctgaaggtgaccaagggtggccccctgcccttcgcctgggacatcctgtcccctcagttcatgtacggctccaaggcctacgtga
agcaccccgccgacatccccgactacttgaagctgtccttccccgagggcttcaagtgggagcgcgtgatgaacttcgaggacggcggcgtggt
gaccgtgacccaggactcctccctgcaggacggcgagttcatctacaaggtgaagctgcgcggcaccaacttcccctccgacggccccgtaatg
cagaagaaaaccatgggctgggaggcctcctccgagcggatgtaccccgaggacggcgccctgaagggcgagatcaagcagaggctgaa
gctgaaggacggcggccactacgacgctgaggtcaagaccacctacaaggccaagaagcccgtgcagctgcccggcgcctacaacgtcaa
catcaagttggacatcacctcccacaacgaggactacaccatcgtggaacagtacgaacgcgccgagggccgccactccaccggcggcatg
gacgagctgtacaagtccggactcagatctcgagctcaagcttcgaattctgcagtcgacggtaccgcgggcccgggatccaccggatctagag

gcggcggctcccatatgtagtgctagcataacttcgtatagtataccttatacgaagttatggcgcgccacatgattataacctgcagggcgcgtgat
cttacggctgcgccttcacgtcggagatgggggtgtgaccctcgcccctcccacccgcgccacggccggatggagaccgcgacggaggcaac
gacgacggcgtgggagggggctcggggcgcgtataaagccatgtgtatgtcatcccaataaagtttgccgtgcccgtcaccatgcccgcgtcgtc
cgtgcgcctcccgctgcgcctcctgaccctcgcgggcctcctggccctcgcgggggccgccgccctcgcccgcggcgcgccgcagggtgggcc
gccctcgccgcagggggggtcccgcgcccaccgcggcgcccgcgcgcgggcccaccctgttcgtcctggacggcgacggctccgcgtggttcgt
cttccagctcggcgggctgggggcgctcaacgacacgcgcatccgcgggcacctgctcggccggtacctcgtctcgtaccaggtggtgcccccg
cccgtctccgcgtggtactttgtgcagcgcccgcgcgagcgcccgcgcctctcggggccgccctcgggcgcggagctcgtggccttcgacgcgc
ccggcgtccggcgcacgtacaccacggcggcggtgtggcccgcggaggtggccgtcctcgcggacgcggag

Supplementary Table1. Oligonucleotide primers used in this study

Primers	sequences	application
gE300-F	cgggatctggacgttcctg	gE gene verification
gE500-R	atgggcggctcgcgccggag	
TK114-F	cgcactctgttcgacacgga	TK gene verification
TK367-R	gctgatgtccccgacgatga	
gEhm1-F	gaacgggaccacggacctct	gEhm1 amplification
gEhm1-loxP-R	atacgaagttattggcagggcctgccgccccggtatctgcgcggcgcgcagcagaaagg	
gEhm2-loxP-F	tatacgaagttatgcggccgcaggaacccctagtaaccgcctgttgatgtcccgccccg	gEhm2 amplification
gEhm2-R	acgcgctcgcgacaactgcg	
TKhm1-F	ccgccttatcatccccgctccccgccgccg	TKhm1 amplification
TKhm1-loxN-R	ttctagaggtacctgacgacacctgaaatggaagggcgccgtcgaggtagatccggagg	
TKhm2-loxN-F	cttacggctgcgccttcacgtcggagatgggggtgtgaccctcgcccctcccacccgcg	TKhm2 amplification
TKhm2-R	ctccgcgtccgcgaggacggccac	
GFP-loxP-F	gattgaattcgggaggaggaggatccgtgagcaagggcgaggagctgttc	GFP amplification
GFP-loxP-R	gaggcaagcttcggatcctcctcctcccttgtacagctcgtccatgccgagag	
mCherry-loxN-F	cggggtggatcctggcggcggctccgtgagcaagggcgaggagga	mCherry amplification
mCherry-loxN-R	agcactacatatgggagccgccgcctctagatccggtggatcccgggccc	
sgRNA-TK-F	caccggtctgcgcatcgtaaatac	TK sgRNA cloning
sgRNA-TK-R	aaacgtatttacgatgcgcagacc	
sgRNA-gE-F	caccgccgtgttctttgtggcggt	gE sgRNA cloning
sgRNA-gE-R	aaacaccgccataaagaacacggc	
